# Strain Transfer for Optimal Performance of Sensing Sheet

**DOI:** 10.3390/s18061907

**Published:** 2018-06-12

**Authors:** Matthew Gerber, Campbell Weaver, Levent E. Aygun, Naveen Verma, James C. Sturm, Branko Glišić

**Affiliations:** Departments of Civil and Environmental Engineering, and Electrical Engineering, Princeton University, Princeton, NJ 08544, USA; gerberbeam@gmail.com (M.G.); campbellweaver@mac.com (C.W.); laygun@princeton.edu (L.E.A.); nverma@princeton.edu (N.V.); sturm@princeton.edu (J.C.S.)

**Keywords:** structural health monitoring, strain transfer, sensing sheet, large area electronics, damage detection, flexible adhesive

## Abstract

Sensing sheets based on Large Area Electronics (LAE) and Integrated Circuits (ICs) are novel sensors designed to enable reliable early-stage detection of local unusual structural behaviors. Such a device consists of a dense array of strain sensors, patterned onto a flexible polyimide substrate along with associated electronics. Previous tests performed on steel specimens equipped with sensing sheet prototypes and subjected to fatigue cracking pointed to a potential issue: individual sensors that were on or near a crack would immediately be damaged by the crack, thereby rendering them useless in assessing the size of the crack opening or to monitor future crack growth. In these tests, a stiff adhesive was used to bond the sensing sheet prototype to the steel specimen. Such an adhesive provided excellent strain transfer, but it also caused premature failure of individual sensors within the sheet. Therefore, the aim of this paper is to identify an alternative adhesive that survives minor damage, yet provides strain transfer that is sufficient for reliable early-stage crack detection. A sensor sheet prototype is then calibrated for use with the selected adhesive.

## 1. Introduction

A critical detail in large area electronics (LAE) for damage detection is the way the sensing sheet is attached to the structure. While previous research has successfully addressed this issue for sensors bonded to less hard materials such as concrete, this research explores the behavior of three new adhesive options for sensors attached to a hard material such as aluminum. The most suitable adhesive is selected by performing both load (non-destructive) and damage tests using individual full-bridge strain sensors. Factors such as set time, strain transfer and adhesive strength are considered in selecting the adhesive. Using the selected adhesive, an LAE strain sensor is calibrated for strain detection.

### 1.1. Strain-Based Monitoring

While there are many parameters that can be monitored to detect structural damage, strain is frequently used because it is directly related to stress (e.g., according to Young’s Modulus). Since materials fail when stress exceeds the ultimate limit state, stress would be the ideal structural parameter to monitor in order to evaluate a structure’s health. However, because there are currently no effective means to monitor stress on a structure under real conditions, strain is selected as the next most suitable parameter. Damage to a structure (e.g., cracking or bowing) frequently creates strain field anomalies that could be reliably detected if the strain sensor is installed in the perturbed area (i.e., in direct contact with or in close proximity to the damage) [1]. The most mature and widely used strain monitoring sensors involve resistive strain gages. A strain change in the structure changes the gage wire length, thus changing the wire’s cross-sectional area and resistance. A change in the wire’s resistance causes a change in voltage drop across the sensor [2].

**Figure 1:**
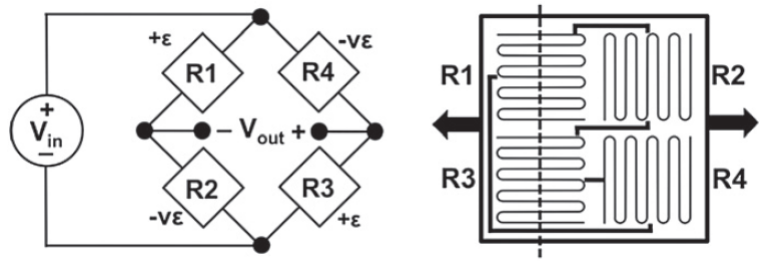
Wheatstone Bridge. The dashed line represents a crack running through the sensor [3].

As an example, a full-bridge resistive strain sensor that contains the Wheatstone Bridge is shown in Figure 1. While this is merely one example of a resistance-based strain sensor, understanding how it works will prove to be helpful in thinking about how sensors translate a change in strain into a quantifiable measurement that allows for damage assessment. See [3] for Equations (1) to (4).

The Wheatstone Bridge consists of four resistors—$R_{1}$, $R_{2}$, $R_{3}$, and $R_{4}$. Resistors $R_{1}$ and $R_{3}$ are oriented parallel to the strain measurement axis, and resistors $R_{2}$ and $R_{4}$ are oriented perpendicular to this axis. The ratio of output voltage ($V_{out}$) to input voltage ($V_{in}$) is expressed in terms of the four resistors and is given by Equation (1):

(1) $$V_{out}=\left(\frac{R_{4}}{R_{3}+R_{4}}-\frac{R_{1}}{R_{1}+R_{2}}\right)V_{in}$$

If each sensing element has the same initial resistance, Equation (1) simplifies to $V_{out}=0$. If a crack occurs, at least one of the four resistive strain sensors is affected, causing a change in resistance, ΔR. From Equation (1) the relationship between change in resistance, ΔR and change in voltage, $\Delta V_{out}$ is given by Equation (2):

(2) $$V_{out}+\Delta V_{out}=\Delta V_{out}=\left(\frac{R_{4}+\Delta R_{4}}{R_{3}+\Delta R_{3}+R_{4}+\Delta R_{4}}-\frac{R_{1}+\Delta R_{1}}{R_{1}+\Delta R_{1}+R_{2}+\Delta R_{2}}\right)V_{in}$$

The gage factor for a resistive element is defined as the ratio of relative change in resistance to relative change in strain: $GF=\frac{\Delta R/R}{\epsilon }$. Equation (2) can be written in terms of gage factor and strain change:

(3) $$\Delta V_{out}=\left(\frac{1+GF\epsilon_{4}}{2+GF\epsilon_{3}+GF\epsilon_{4}}-\frac{1+GF\epsilon_{1}}{2+GF\epsilon_{1}+GF\epsilon_{2}}\right)V_{in}$$

In the case of uni-axial stress, as shown in Figure 1, resistive elements $R_{1}$ and $R_{3}$ experience full strain because they are oriented parallel to the strain axis, but elements $R_{2}$ and $R_{4}$ also experience strain according to Poisson’s ratio, ν. Finally, we arrive at an equation for strain change in terms of voltage ratio ($\Delta V_{r}=\frac{\Delta V_{out}}{V_{in}}$), gage factor (GF) and Poisson’s ratio (ν), given by Equation (4):

(4) $$\epsilon =\frac{-2\Delta V_{r}}{GF[\left(\nu +1\right)-\Delta V_{r}\left(\nu -1\right)]}$$

An important property about the full bridge sensor is its insensitivity to temperature change. A change in temperature of the sensor, ΔT causes a change in temperature in each of the four resistive elements: ΔT = $\Delta T_{R1}$ = $\Delta T_{R2}$ = $\Delta T_{R3}$ = $\Delta T_{R4}$, because all four resistive elements are close to each other. A change in temperature, ΔT causes a change in resistance, $\Delta R_{T}$, according to the equation: $\Delta R_{T}=R_{o}\alpha \Delta T$ , where $\Delta R_{T}=\Delta R_{T1}=\Delta R_{T2}=\Delta R_{T3}=\Delta R_{T4}$. Since the resistance in each resistor after manufacturing is assumed to be the same, $R=R_{1}=R_{2}=R_{3}=R_{4}$, Equation (2) yields $\Delta V_{out}=0$, so a change in temperature does not cause a change in voltage. Therefore, temperature compensation is not necessary in this type of sensor.

### 1.2. 2D Strain Monitoring

**Figure 2:**
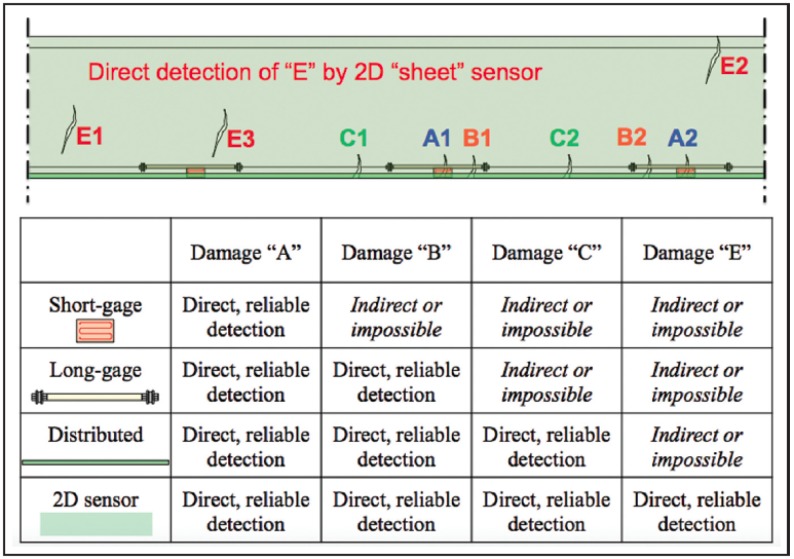
Short-gage, long-gage, distributed, and 2D sensor damage detection capabilities [1].

Besides resistive strain sensors, there are several other types of strain sensors, based either on the physical principle behind measurement (e.g., vibrating wires, fiber optics, etc.) or on the spatial extent of sensors. Based on the spatial extent of sensors, the sensors could be discrete, short-gage, long-gage, distributed, one-dimensional (1D), or two-dimensional (2D) [1]. Regardless of the type or spatial extent, strain sensors that are in direct contact with the damage-induced strain-field anomaly can detect this anomaly reliably, as the anomaly will cause an unusually high change in output signal of the sensor [4]. Nevertheless, the spatial extent of the sensor plays an important role in increasing or decreasing the likelihood of detection of strain-field anomalies (i.e., of damage). Figure 2 demonstrates the damage detection capabilities for short-gage, long-gage, distributed and two-dimensional sensors. As expected, the distributed sensor performs the best among the three one-dimensional options, but is unable to detect damage of type "E", since the damage lies out of reach for the sensor. The 2D sensor, on the other hand, promises to reliably detect all four types of damage illustrated in this example, as it is in direct contact with each of them.

### 1.3. 2D Strain Sensing Research and Testing

Discrete, long-gage, and short-gage sensors, as well as 1D distributed sensors are commercially available, which is not the case for 2D distributed sensors. Great promise for reliable damage detection motivates the need for research in that area. Hence, several researchers have explored 2D sensors in various forms of sensing skins [5,6,7,8,9], expandable sensor networks [10], nano-paints, and nano-based materials and adhesives [11,12,13,14], photonic crystals [15], etc.

Several researchers from Princeton University jointly created a 2D sensor called sensing sheet, based on Large Area Electronics (LAE) and Integrated Circuits (ICs) [1,2,3,16,17,18,19,20]. LAE enables the integration of dense arrays of diverse sensors and accessory electronics onto thin, deformable plastic substrates, which can cover large areas of structures and enable direct damage [21,22,23,24,25]. Sensing sheets have three main components [16]: (1) a two-dimensional dense array of unit strain sensors patterned on a polyimide (Kapton) substrate and combined with functional LAE; (2) embedded ICs interfaced via non-contact links for sensor readout, data analysis, power management, and communication; and (3) an integrated flexible photovoltaic sheet and power converters (rechargeable batteries) on the LAE sheet to power the full system and protect it from elements (wind, rain, snow, ultraviolet radiation, etc.).

**Figure 3:**
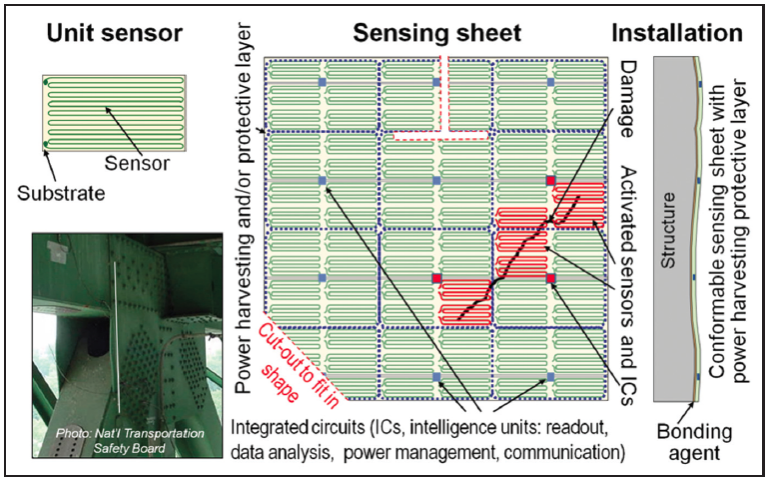
Schematic representation of the sensing sheet based on LAE and ICs [3].

It is important to note that while the general sensing principle of sensing sheet is based on strain measurement, its purpose is not to accurately measure strain magnitudes, but rather to detect, localize, and quantify unusual behaviors that manifest in the form of strain-field anomalies (e.g., cracking of concrete, fatigue cracking, and bowing of steel). Being in direct contact with sensing sheet individual sensors, the strain-field anomalies create unusually high strain changes in these sensors compared with individual sensors that are not in direct contact with the anomaly. This unusually high strain change is used to directly detect and localize the anomaly. The extent of the anomaly is then inferred from geographical locations of affected individual sensors. This principle is shown in Figure 3.

The applications of sensing sheet could be on a variety of civil and mechanical structures and infrastructure, including, but not limited to: bridges, buildings, tunnels, pipelines, wind turbines, aircrafts, etc. Given that accurate measurement of strain is not required, the imperfections and phenomena that affect measurements in the range of usual strain changes (e.g., potential rheological strain in glue, lower strain transfer due to gluing over the paint of original structure, etc.) would not significantly affect the performance of the sensing sheet for this function. More detail is given in literature [1,2,3,16,17,18,19,20].

Yao and Glišić [16] performed preliminary tests to evaluate whether an early-stage LAE prototype has the potential to detect damage, such as a crack [1]. The authors tested a full-bridge resistance-based strain sensor as a preliminary step towards LAE.

Series of tests were performed at the Carleton Laboratory at Columbia University on an LAE-based strain sensing sheet prototype in order to evaluate the sensor’s ability to detect cracks in steel plate specimens [16]. In these tests, the sensors were bonded to the steel samples with a notch, and were exposed to fatigue cycling, so that the crack occurrence and propagation could be predicted. While the tests were successful from the point of view of damage detection, they revealed a challenge: individual sensors would fail immediately upon damage occurrence, thereby limiting the ability of sensing sheets to follow and quantify damage progression. That challenge is addressed in this paper.

## 2. Selecting a Suitable Adhesive

### 2.1. Objective

Because manufacturing sensing sheets at the prototype stage is expensive, the following tests are performed using existing identical strain-gages.

**Figure 4:**
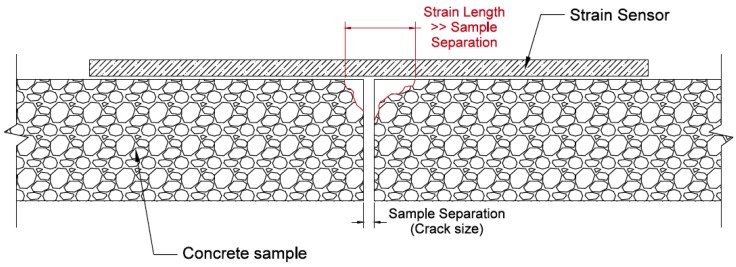
Strain length vs. sample separation on degrading materials. Degradation is indicated by red lines.

**Figure 5:**
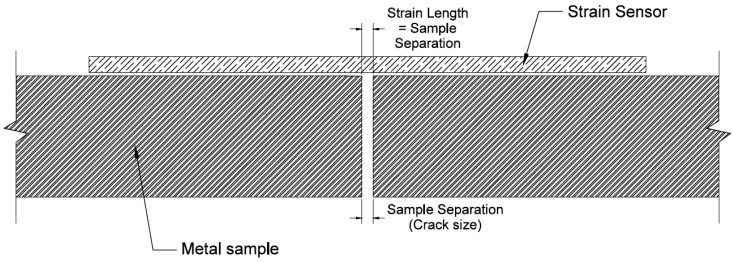
Strain length vs. sample separation on non-degrading materials.

A critical component of a sensor’s functionality is the way the sensor is fixed to the structure’s surface. Because the sensing sheets involved in this study are formed on a polyimide substrate, an adhesive is the best fixing option. At minimum, a suitable adhesive should maintain bond between the sensor and structure under various loads, damage types, and environmental conditions. An equally important consideration involves the adhesive’s elastic behavior, particularly in materials that do not mechanically degrade when damaged. Due to the heterogeneous nature and particle flaws, concrete degrades when subjected to tensile stresses. When subjecting a strain sensor bonded to concrete to moderate strains, the bond between the sensor and concrete is stronger than the bond between aggregate particles in the concrete, causing the concrete to break apart near the crack. In this case, the strain in the sensor is defined over the strain length, shown in Figure 4. Conversely, when subjecting a strain sensor bonded to non-degrading materials, such as aluminum and steel, the bond between particles in the material is much stronger than the bond between the sensing sheet and structure, so the strain is defined over the sample separation (See Figure 5), leading to much higher strain values. Mechanical strain in the sensor is calculated as the change in length divided by original length. For a perfectly non-degrading material that has no initial separation, any separation will theoretically cause infinite strain because original separation length is zero. Hence, in non-degrading materials the sensor will fail soon after crack occurrence, which in turn will disable monitoring of crack size and its evolution. Therefore, the purpose of this study is to identify and both qualitatively and quantitatively evaluate an adhesive to be used for two-dimensional sensing on non-degrading materials, so that the sensor (sensing sheet) survives crack occurrence and continues monitoring the crack size.

The soft adhesives considered in this research are expected to have viscoelastic behavior, and consequently, rheological effects in adhesives may influence values of strain measurements over time. However, as the main aim of this research was to identify an adhesive that enables the sensing sheet to survive the damage to the structure, only short-term effects were analyzed, and long-term effects will be a subject of future work. This approach was considered suitable as the maximum stress in sensing sheet happens instantaneously, with occurrence of damage (rheological effects are actually beneficial as they bring relaxation). In addition, rheological effects are small compared to strain changes due to damage, and thus they do not significantly affect its damage detection capability (they affect strain measurement, but accurate strain measurement is not the purpose of the sensing sheet).

### 2.2. Qualitative Tests

**Figure 6:**
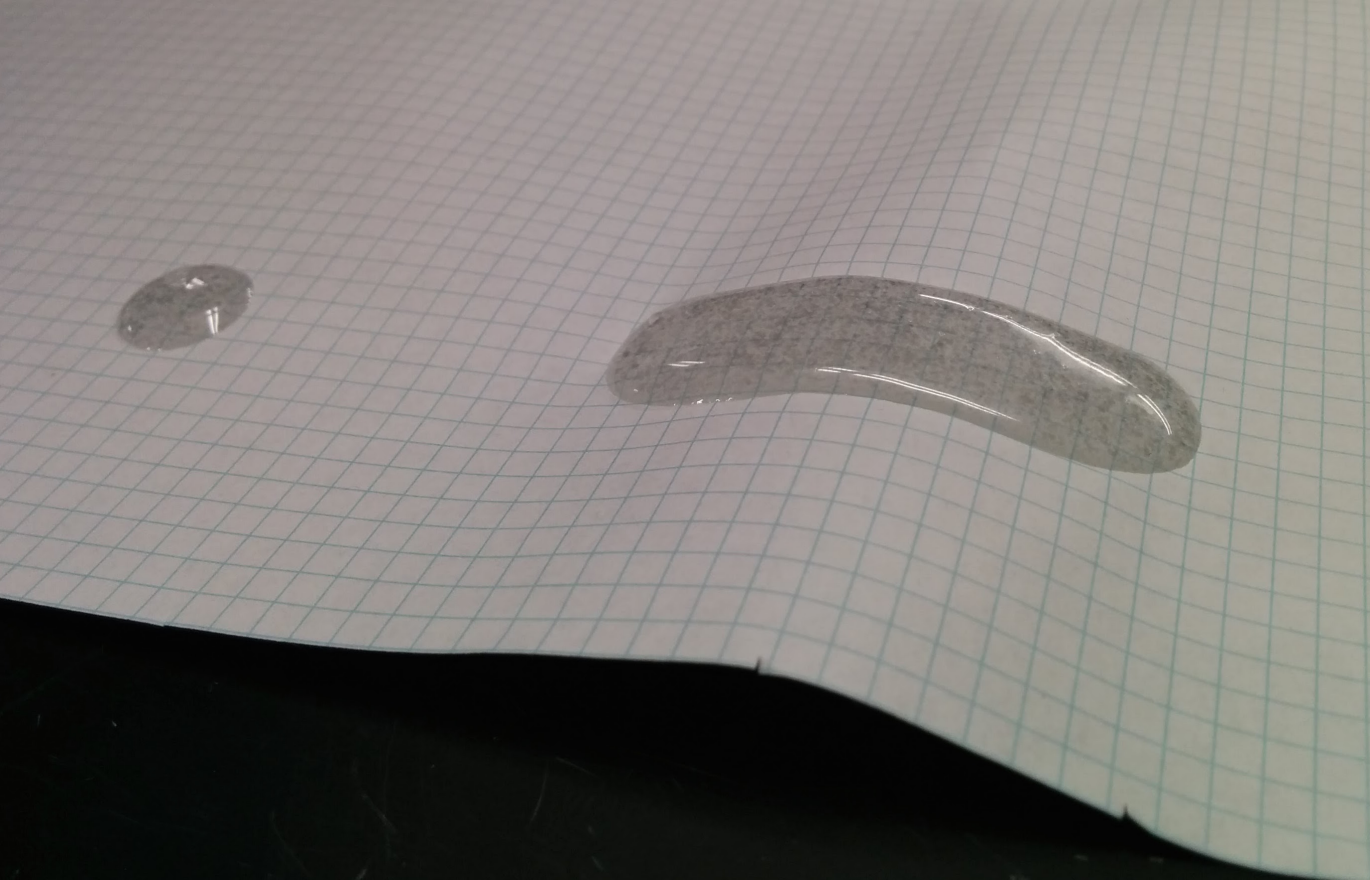
Sample of a flexible adhesive applied to paper.

Qualitative observations were performed for three high-strength flexible adhesive alternatives: 3M DP100, DP105 and DP125. Initially, these three adhesives were tested on paper samples in order to gain an understanding of viscosity, flexibility and set time. It was found that the three adhesives listed were very flexible and could be bent and twisted (along with the substrate paper sheet) by hand without much effort. A sample of a flexible adhesive applied to paper and bent can be seen in Figure 6, below.

The 3M DP100 had a set time of about five minutes, the DP105 had a set time of about 20 minutes, and the DP125 had a set time which exceeded half an hour. An ideal set time is neither too short (to give adequate time to place the sensor sheet) nor too long (to avoid sensor displacement caused by accidental contact shortly after placement). A set time exceeding a half hour is undesirable because it increases the risk of accidentally moving or removing the sensor during the installation phase. Because the DP125 adhesive set time exceeded half an hour, it was eliminated, and the DP100 and DP105 adhesives were further tested.

### 2.3. Quantitative Tests: Loading and Unloading

**Figure 7:**
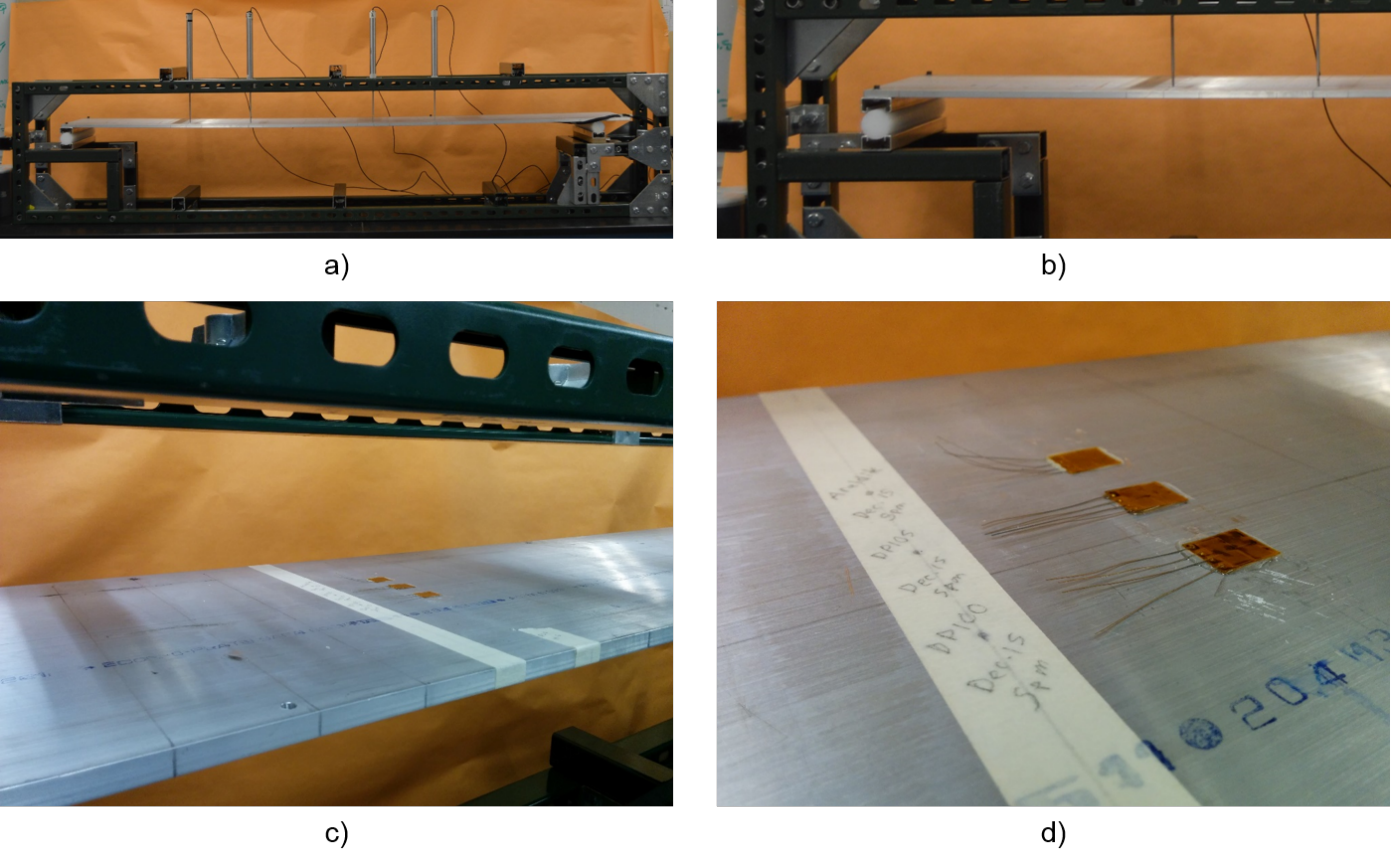
Strain transfer test set-up: (**a**) view of beam without sensors; (**b**) support detail; (**c**) view of sensors installed using Araldite 2012, 3M DP 100, and 3M DP 105; (**d**) sensor details.

Strain tests were performed in order to understand how much strain is transferred to the sensor from a monitored material given that the adhesives are soft and some loss of strain transfer is expected. The test involved bonding three sensors to the top of a thin aluminum simply supported beam and loading and unloading the beam with known point forces. Due to limitations of available testing set-up, bottles filled with sand were used to load the beam. The beam was simply supported at both ends as shown in Figure 7. It had span of 1710 mm, thickness of 9.13 mm and width of 254 mm. The beam was made of aluminum, and had a Young’s modulus of 68 GPa. Strain gages were placed at 855 mm from the left support (in the middle of the beam). The sand bottles B1, B2 and B3 had masses 594.2 g (5.8 kN), 598.1 g (5.9 kN) and 597.7 g (5.9 kN), respectively. For each load step, the strain value was recorded with an Omega DP41-S reading unit (Omega Engineering, Stamford, CT, USA). The total loading and unloading time was less than 2 minutes. The three tested adhesives were: a stiff Araldite 2012 adhesive (used by Yao and Glišić in previous studies [16]), 3M DP100, and 3M DP105. The stiff adhesive was used for reference purposes, as it is assumed to have high quantity of strain transfer due to its high stiffness. The aluminum beam and three bonded sensors used in the test are shown in Figure 7, below.

**Figure 8:**
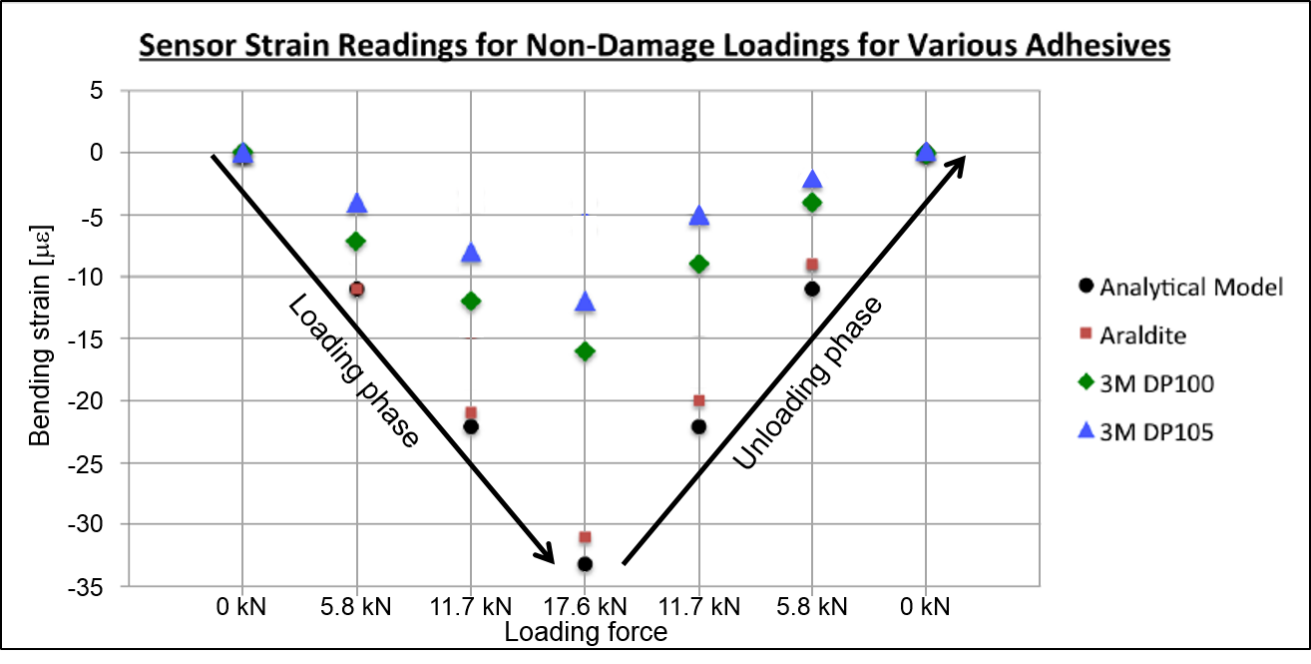
Strain transfer results for various adhesives.

The results of the strain transfer tests are shown in Figure 8. An analytic strain model was developed for the aluminum beam. The model was based on the linear theory of beams and is presented in Equation (5).

(5) $$\epsilon_{s}=\frac{M\times y_{s}}{EI}$$

where: $M=FL^{2}/4$—bending moment at location of the sensors (F is force applied using bottles placed in the middle of the beam, L=1710 mm is length of the beam)

E=68 GPa—Young’s modulus of aluminum

$I=bh^{3}/12$—moment of inertia of the beam’s cross-section (b=254 mm, h=9.13 mm), and $y_{s}=h/2+0.2$ mm—distance of sensors from the horizontal principal axis of the cross-section (where 0.2 mm accounts for estimated thickness of the glue plus half the thickness of the strain gage).

Figure 8 shows the instantaneous bending strain for seven load steps. The first and last load steps correspond with no beam loading, the second and sixth load steps correspond with the beam loaded with sand bottle B1, the third and fifth load steps correspond with the beam loaded with sand bottles B1 and B2, and the fourth load step corresponds with the beam loaded with all three sand bottles. As expected, the stiff Araldite adhesive consistently transfers the greatest fraction of strain from the structure to the sensor, and none of the adhesives transferred all strain to the sensor. The strain values measured by sensors glued with Araldite were about 2.5 times greater than those measured by the other two glues. Figure 8 shows that while DP100 transfers slightly more strain than DP105, in general there is no significant strain transfer difference between the 3M DP100 and 3M DP105 adhesives. Both adhesives “lose” more than 50 percent of strain; however, this is acceptable as the main purpose of the sensing sheet is damage detection and not strain monitoring. Moreover, linear response of both adhesives indicates that lack in strain transfer can be accounted for through calibration and gage factor adjustments. This is effectively done in Section 3 of this paper, where strain transfer was tested on sensing sheet prototypes.

### 2.4. Damage Tests: Crack Opening vs. Strain Test

**Figure 9:**
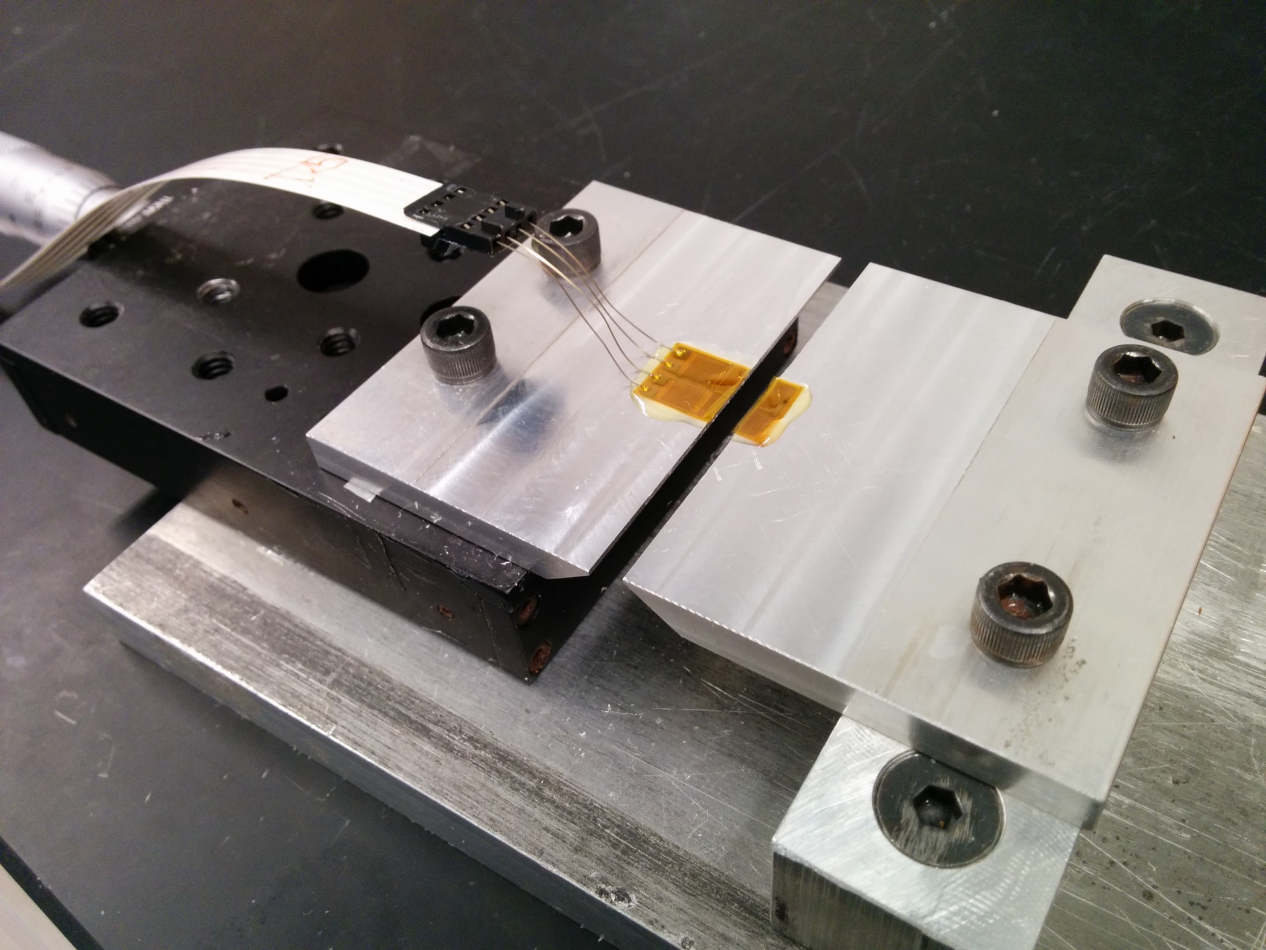
Crack test setup.

The aim of the damage test was to assess the behavior of adhesives at crack locations and to see how it affects the survival of the sensors. For these tests, a sensor was bonded to “closed” aluminum plates, and the adhesive was given time to cure. Once the adhesive reached suitable strength, an artificial crack was generated by pulling the aluminum plates apart, thereby creating a gap between them. The test setup is shown in Figure 9. Each simulated crack opening increment involved pulling the aluminum plates apart by 0.001 inches, taking the strain reading, and then waiting five minutes to record the “relaxed” strain reading. Relaxation is expected due to creep in sensor polyimide substrate as well as due to creep in the adhesive exposed to high stress levels. This process was repeated until failure (i.e., until the sensor could no longer read the strain due to rupture or the strain values began to decrease due to debonding or the adhesive breaking). The tests were performed with Vishay 3800 strain reading units, and the gage factor was calibrated to the manufacturer’s specifications. An example of post-test damage is shown below in Figure 9.

Based on the manufacturer’s specifications, both adhesives achieve 80% of their full strengths if cured for 20 minutes at 23 ${}^{\circ }$C, and they achieve full strength if cured for 48 hours at 23 ${}^{\circ }$C. Given that the temperature of the lab in which the tests were made was set to 17 ${}^{\circ }$C, and could not be brought up to 23 ${}^{\circ }$C, it was decided to perform one test before the full strength was achieved, and one after the full strength was achieved. For practical reasons, the former was performed 24 hours after gluing and the latter 72 hours after gluing (one day longer than specified time of 48 hours, in order to account for lower curing temperature).

**Figure 10:**
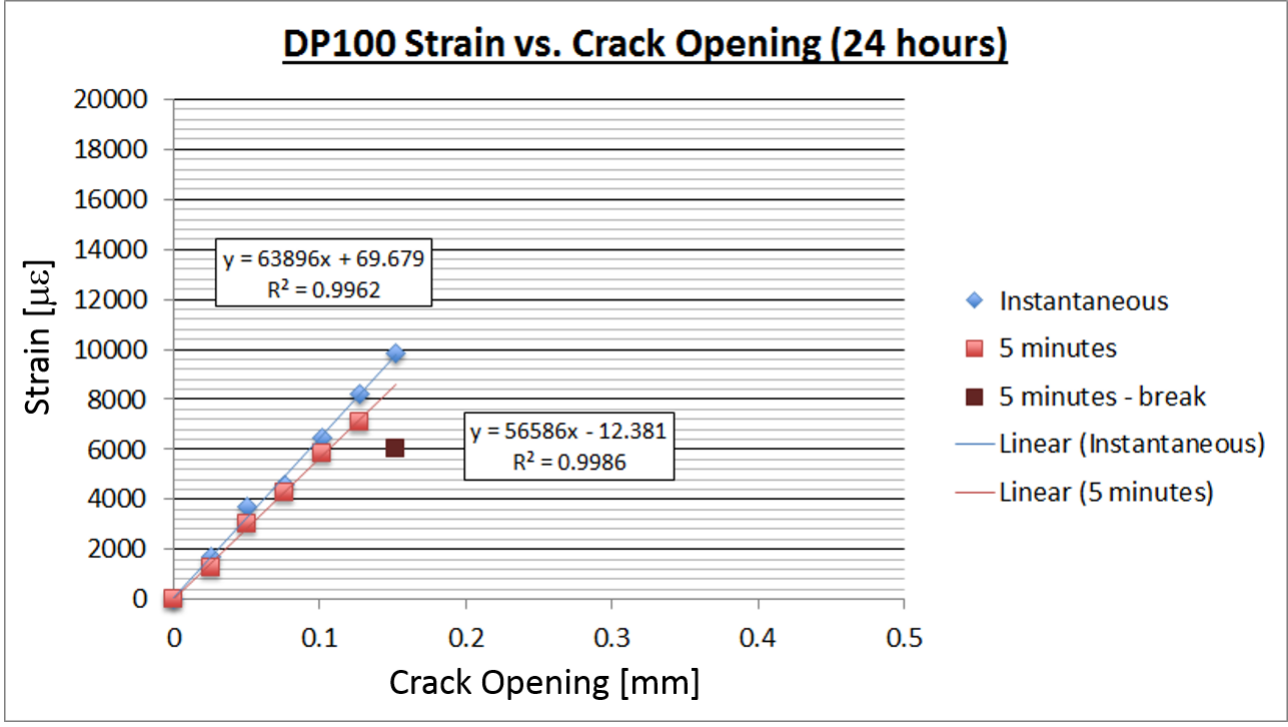
DP100 crack opening vs. strain after 24 hours.

**Figure 11:**
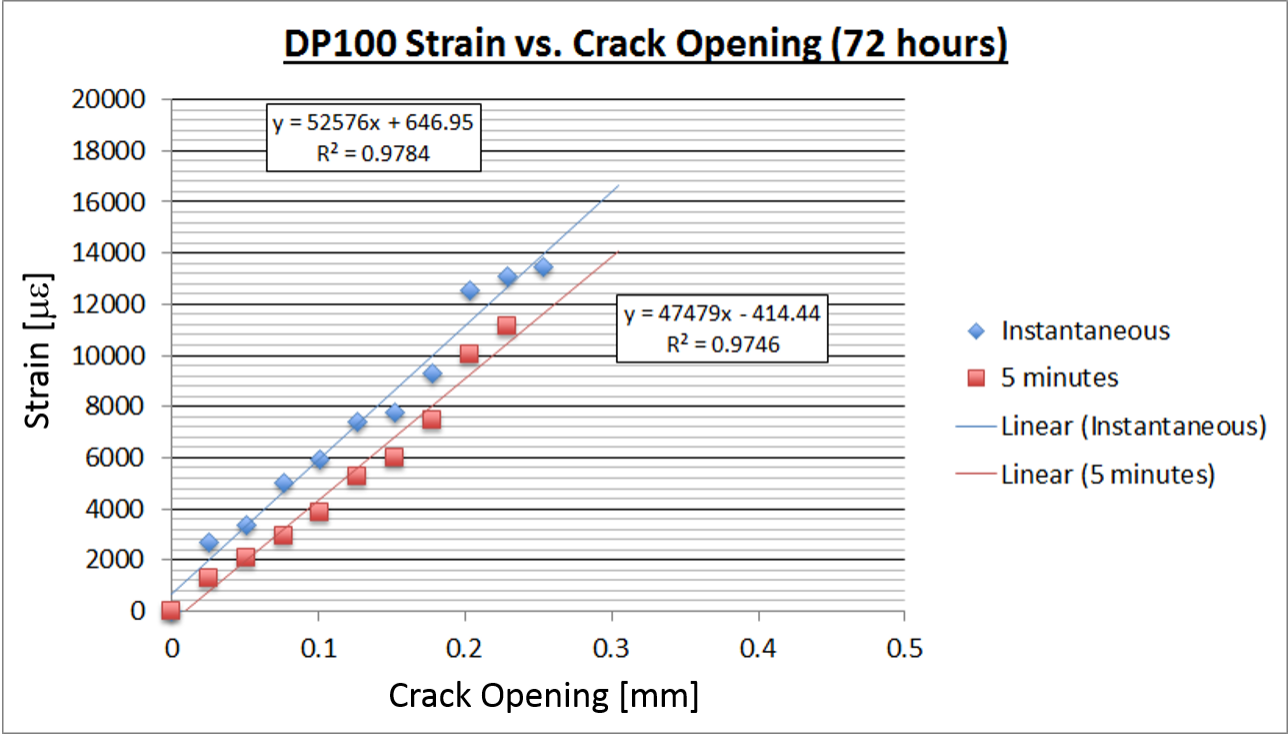
DP100 crack opening vs. strain after 72 hours.

The following plots show the relationship between crack opening size and strain reading for the 3M DP100 adhesive. Both of these tests were performed with strain gages with a gage factor of 2.01. In the first test (Figure 10) the adhesive was allowed to set for 24 hours, and in the second test for 72 hours (Figure 11). Time-dependent tests were performed in order to evaluate the adhesives’ performance with time and to establish a minimum cure time for future lab tests.

The time-dependent tests for the 3M DP100 adhesive showed very interesting results. The tests demonstrated that the bond strength does show time-dependence. The 24-hour DP100 test showed readings up to a crack opening of about 0.15 mm, whereas the 72-hour test showed readings up to a 0.26 mm crack. The result is important because it verifies that the adhesive performs better when given a few days to cure.

**Figure 12:**
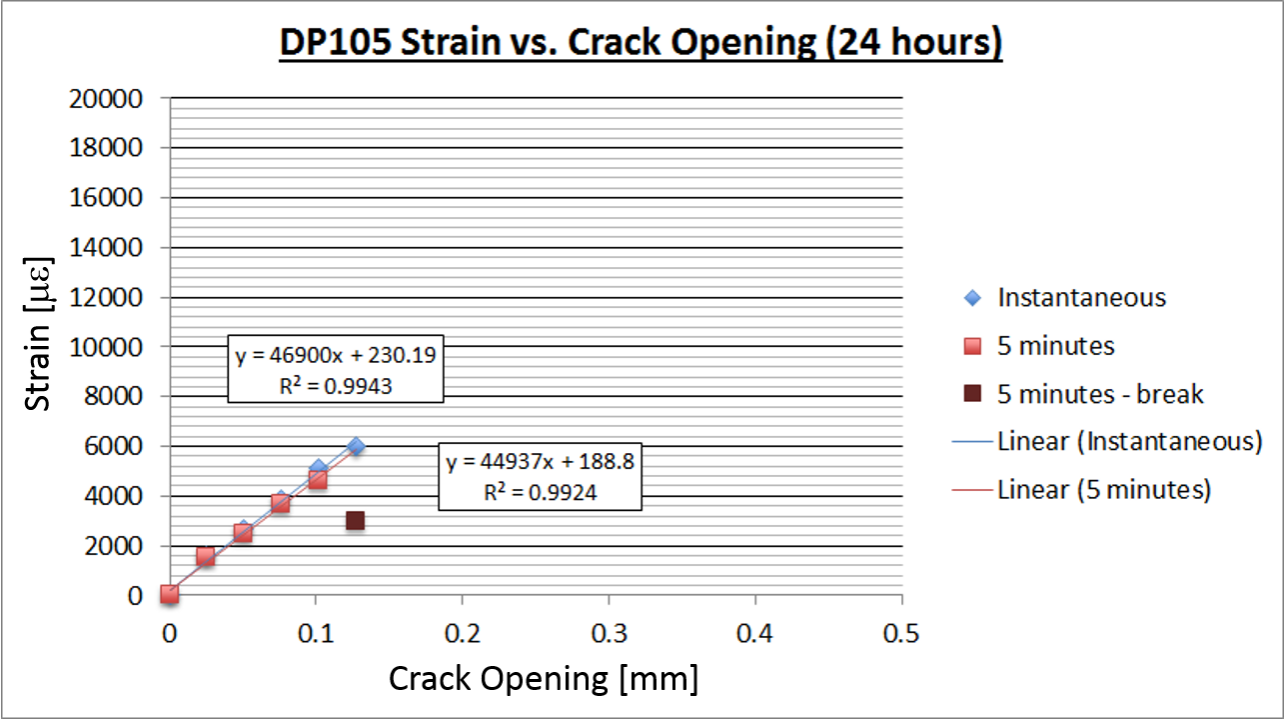
DP105 crack opening vs. strain after 24 hours.

**Figure 13:**
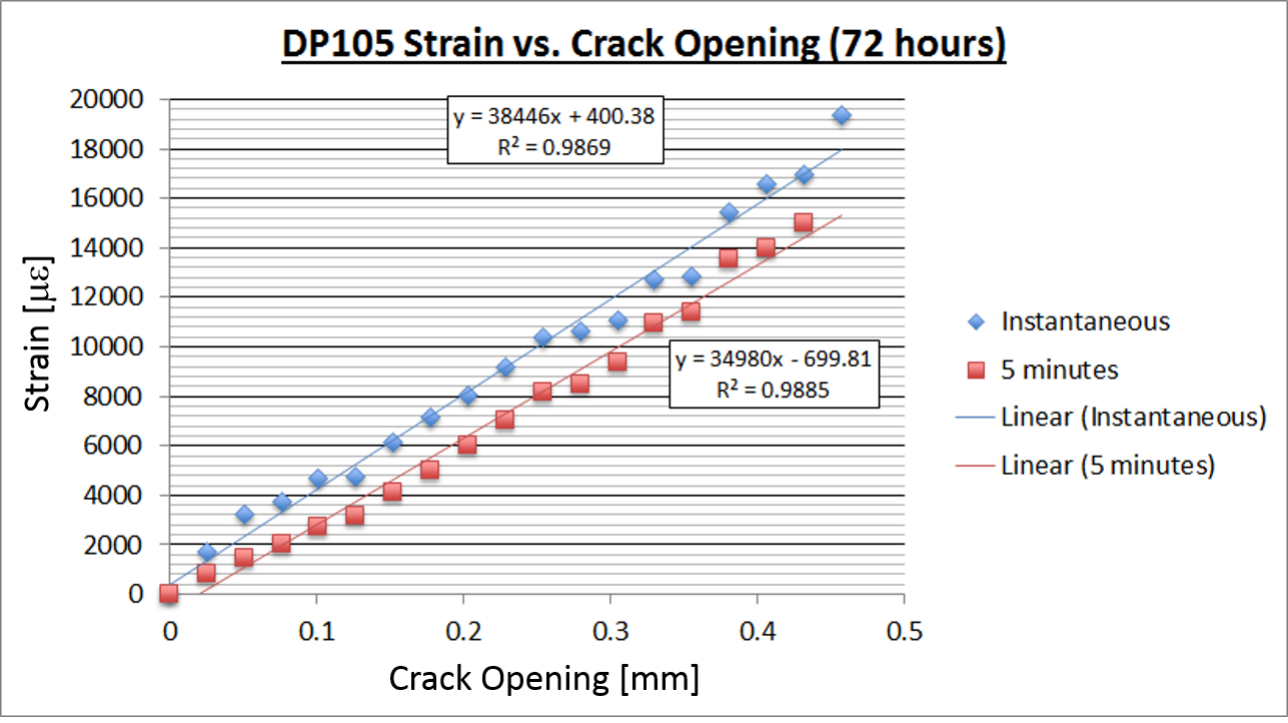
DP105 crack opening vs. strain after 72 hours.

Similar tests were performed for the 3M DP105 adhesive. The results are shown in Figure 12 and Figure 13, below.

The DP105 adhesive continued to read strain for crack openings up to 0.45 mm, compared to about 0.26 mm for the DP100 adhesive. Taking into account that all other properties of the two adhesives are similar, the DP105 adhesive is considered the more suitable adhesive for the installation of sensing sheets that are supposed to detect and survive structural damage.

## 3. Application to a Sensing Sheet Prototype

### 3.1. Objective

Once the appropriate adhesive for the sensing sheet was selected, its application was tested on a sensing sheet prototype. Given that manufacturing of a sensing sheet at the prototype stage is expensive, it was decided to perform only non-damage strain transfer tests, so that the sensing sheet would not be damaged and could be used for future research related to hardware.

The first step in implementing the sensing sheet is to determine appropriate gage factors of the individual sensors and observe how these gage factors change depending on the adhesive used to bond the sheet. The same testing setup as described in Section 2.3 was used. The sensing sheet was installed in the third quarter of the beam, i.e., 1282 mm from the left support. The load was applied 1069 mm from the left support.

**Figure 14:**
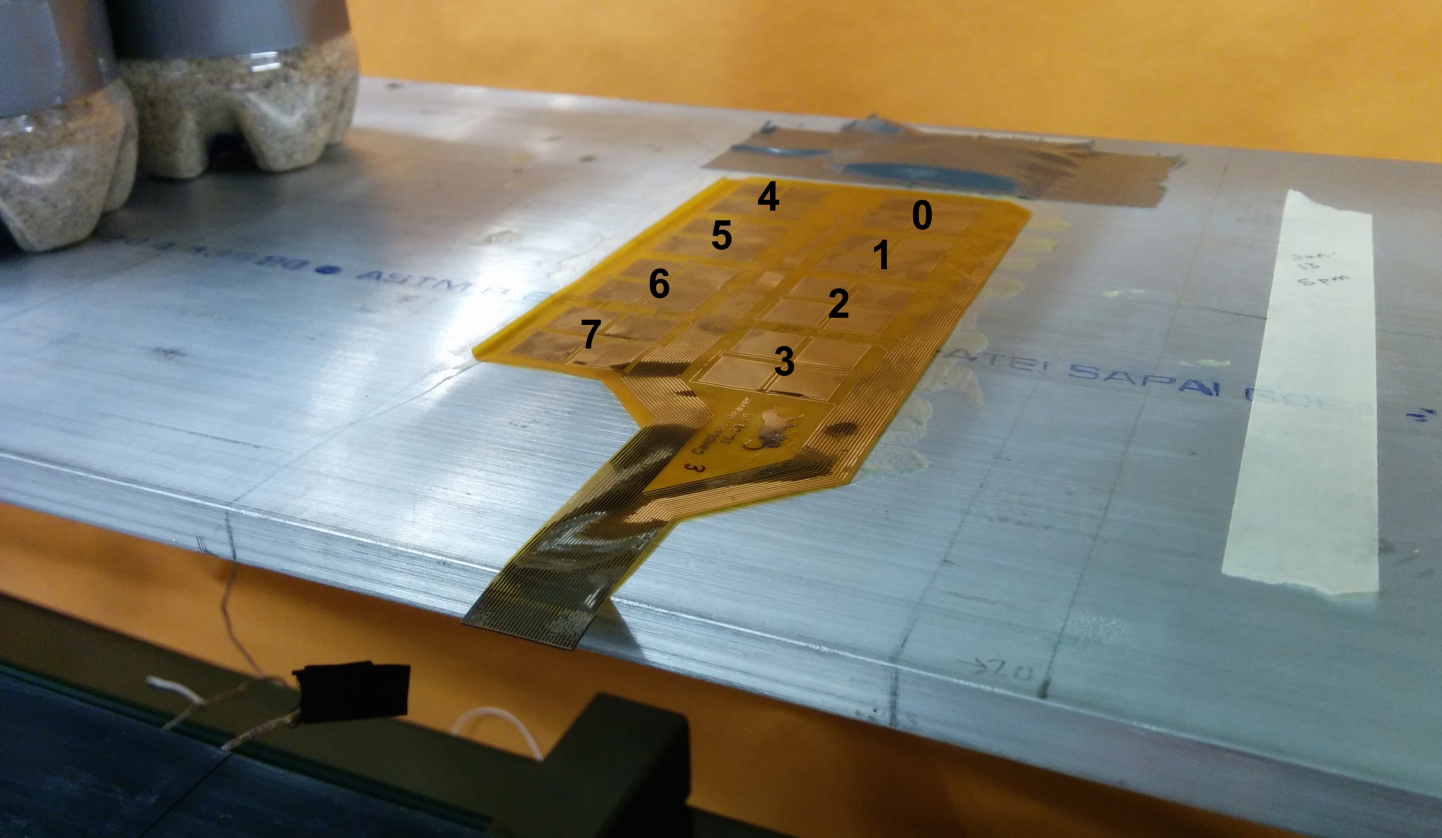
Sensing sheet, and individual sensor numbering convention.

**Figure 15:**
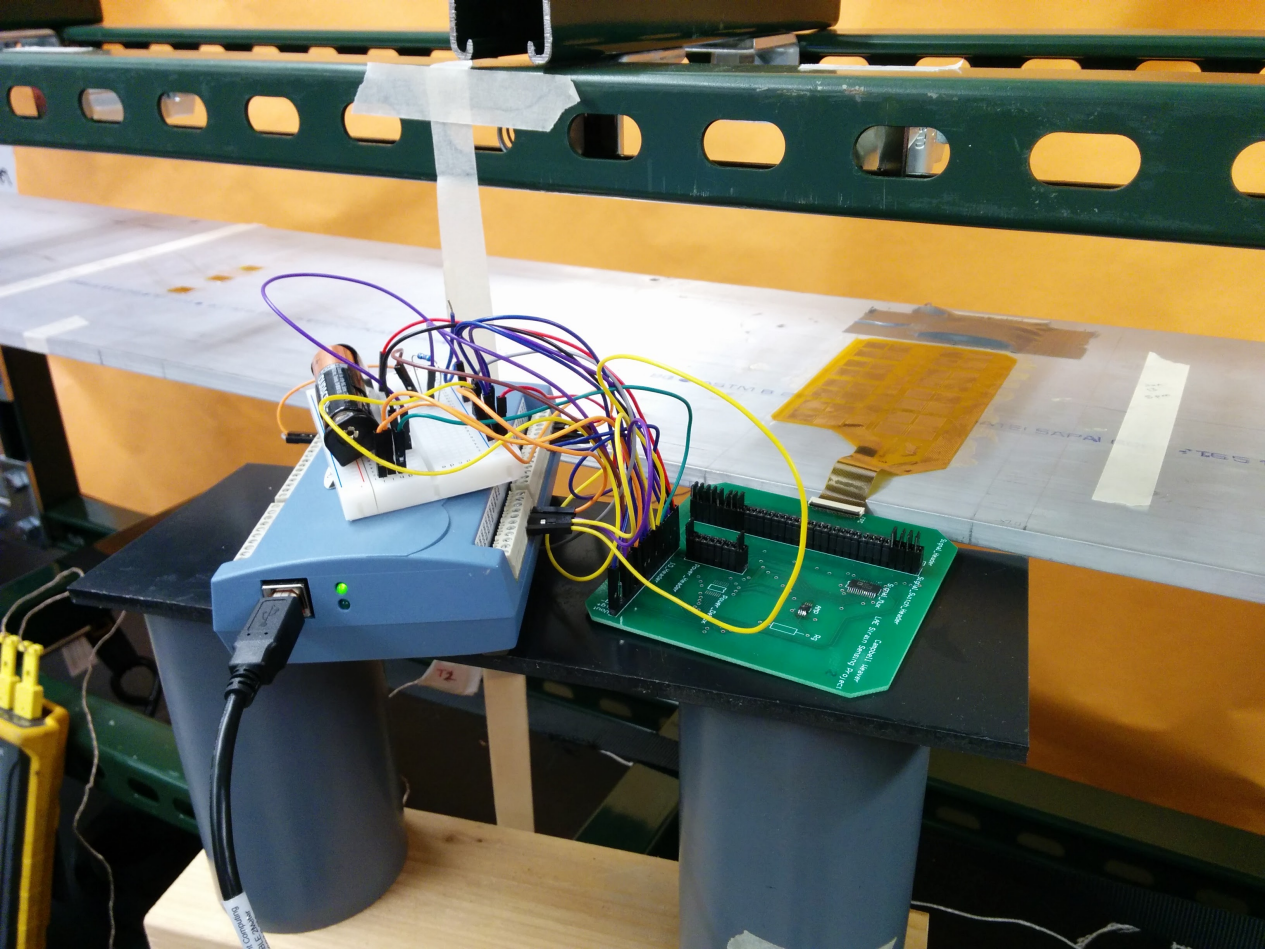
Gage factor test setup. The sensing sheet is bonded to the top surface of the aluminum beam and is connected to the rigid printed circuit board. The temperature reading device is shown in the bottom left corner.

The prototype of the sensing sheet is composed of eight individual sensors, each composed of four resistors forming a full bridge. Each individual sensor was independently tested by bonding the sensing sheet to an aluminum beam, then loading and unloading the beam with sand bottles, similar to the strain-transfer test described in the previous section. In total, two sensing sheets were tested, the first glued with the stiff Araldite adhesive for reference purposes, and the second glued with the soft DP105 glue. Each loading step lasted 15 s, and values were sampled at 1000 Hz, 10 seconds into each loading step. The beam was loaded for seven loading steps: no load, B1, B1 and B2, B1 and B2 and B3, B1 and B2, B1, and no load, giving a total testing time of 105 seconds. At each loading step the change in output voltage was recorded. Using the change in output voltage, the input voltage, and an analytical strain model, the gage factor was determined for each sensor under each loading condition. For each sensor, identical tests were performed three times, yielding three gage factors. Preliminary tests included air and beam surface temperature measurements, but no significant temperature changes were found during the test duration. Figure 14 and Figure 15 show the test setup.

### 3.2. Results

**Figure 16:**
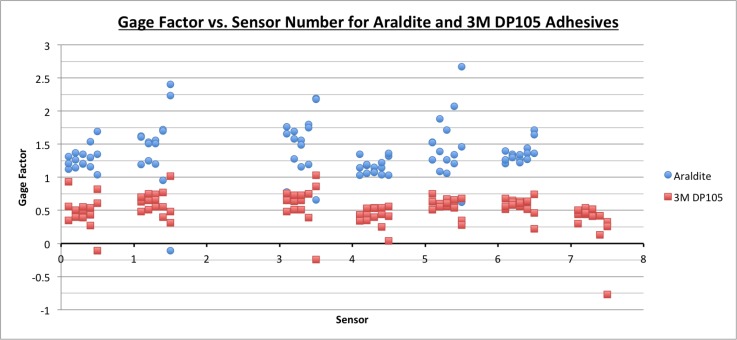
Apparent gage factors determined on individual sensors of sensing sheet prototypes glued with Araldite and 3M DP105 adhesives.

For each loading step for each individual sensor, apparent gage factors were determined under identical loading conditions, and they are shown in Figure 16. Note that these gage factors are apparent, and not true, as they combine true gage factor with strain transfer quality. The figure shows that consistent results are obtained for all individual sensors within the same sheet. In addition, the gage factors for sensors attached using the DP105 adhesive are about 2.5 times less than those for the sensors attached using Araldite (see Table 1). This result is consistent with strain transfer test results described earlier in Section 2.

**Figure 17:**
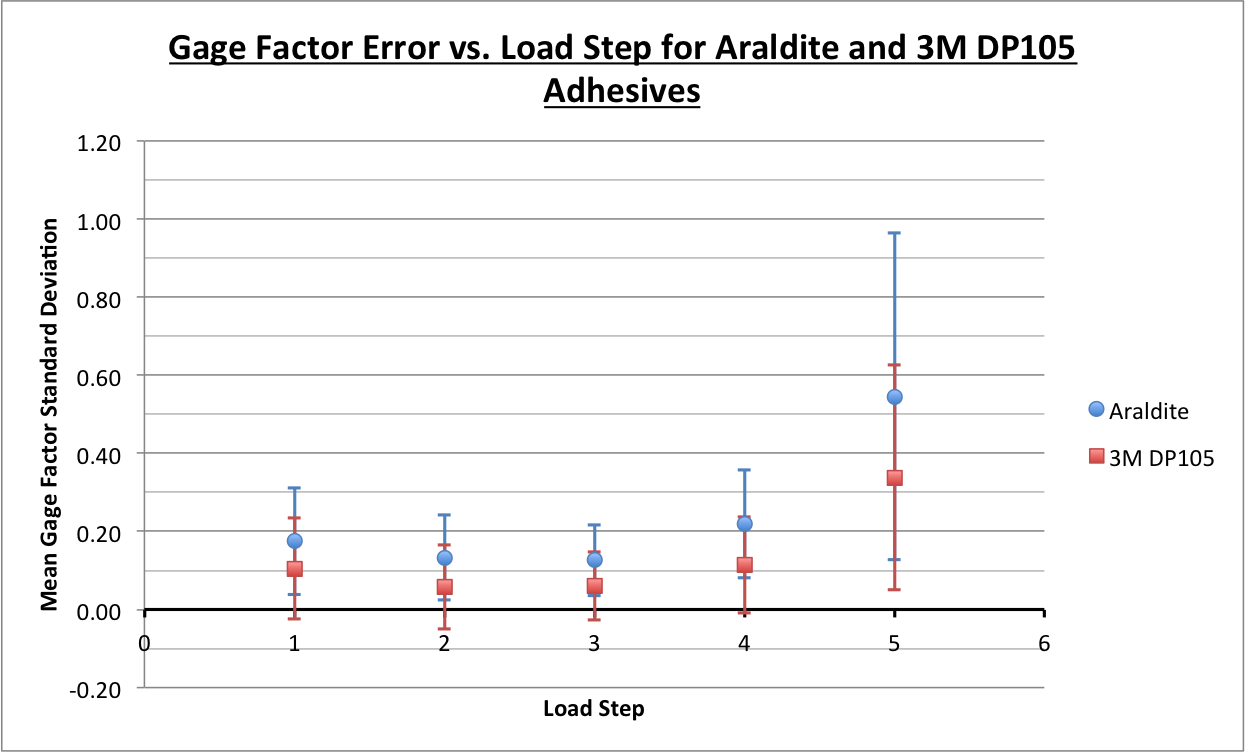
Apparent gage factor uncertainties for various load steps.

As expected, smaller loads yielded greater gage factor errors than large loads due to lower signal to noise ratios. This created dispersion in the results. Figure 17 plots the gage factor uncertainty, measured as standard deviation for each load step. Indices 1 and 5 represent the beam loaded with bottle B1, 2 and 4 represent loading with bottles B1 and B2, and 3 represents loading with all three bottles. Loading step 3 has the smallest gage factor uncertainty, with a standard deviation of about 0.06 for 3M DP105 and 0.13 for Araldite. Loading step 5 has the greatest gage factor uncertainty, with a standard deviation of about 0.33 for 3M DP105 and 0.54 for Araldite. As Figure 17 shows, the Araldite and 3M DP105 have similar gage factor uncertainty trends, except the Araldite’s gage factor uncertainties are consistently greater than those for 3M DP105. The reason for this could be the imperfections and connection issues in the flexible to rigid interface where a zero-insertion-force (ZIF) connector is used. Unwanted impedance in this connection can vary by insertion and introduces a small amount of noise into the differential voltage, which is used to calculate strain. This also shows the need for an integrated system implementation on a flexible substrate and the need to reduce the number of interfaces to rigid boards as mentioned in previous works [18,19,20].

### 3.3. Discussion

The apparent gage factor for Araldite is greater than the apparent gage factor for 3M DP105 adhesive because the Araldite is less flexible and transfers more strain from the structure to the sensor. Strain is proportional to change in output voltage and inversely proportional to gage factor, so if an analytical strain model is used to determine gage factor, a stiff adhesive will result in a larger change in output voltage, and the gage factor will be higher. Obtained results are consistent with the results of the strain transfer test presented in Section 2.

For the sensing sheet bonded with Araldite, sensors 2 and 7 showed high voltage output noise in the change in output voltage values, and therefore did not yield reasonable gage factor values. For the sensing sheet bonded with 3M DP105, sensor 2 had high noise and did not yield reasonable gage factor values. Because change in output voltage values for sensor 2 were unreasonable in both the Araldite and 3M DP105 tests, it is likely that there is a design or manufacturing flaw in the sensing sheet itself, or a soldering problem on the rigid printed circuit board (see Figure 15). In the case of sensor 7 in the Araldite-bonded sensor sheet, the high noise can likely be attributed to a manufacturing flaw on the sensor sheet itself.

Figure 16 shows that there is a range in apparent gage factor values (dispersion) from sensor to sensor. One reason the gage factors might vary from sensor to sensor has to do with a possible voltage drop from the sensor to the rigid printed circuit board and reading unit. Because the wires in the sensing sheet have high resistance, sensors that are located farthest from the reading unit (sensors 0 and 4, for example) may show a lower voltage output change than sensors that are closer to the reading unit (sensors 3 and 8). Furthermore, a lower voltage output change will result in a lower gage factor. Figure 16 shows generally lower gage factors for sensors 0 and 4, validating this hypothesis. Sensors 1 and 5 show higher gage factors and exhibit similar behavior. Sensor 7 does not follow the trend described, which may be attributed to a manufacturing flaw. Another reason for varying gage factors between sensors is that the adhesive layer’s thickness might vary across the sensor sheet. A thinner adhesive layer will permit more strain to be transferred to the sensor, and the gage factor will be higher. Conversely, a thicker adhesive layer will transfer less strain to the sensor and will result in a lower computed gage factor.

Finally, Figure 17 and Table 1 show that the Araldite-bonded sensing sheet yields greater gage factor uncertainties than the 3M DP105-bonded sensing sheet. The Araldite adhesive is much more viscous than the 3M DP105 adhesive and did not spread as easily when bonding the sensor sheet to the aluminum beam, resulting in minor air pockets under the sensor sheet. With the 3M DP105 adhesive it was much easier to spread an even layer on the aluminum beam, and no air pockets were visible, likely increasing apparent gage factor consistency both between and within sensor sheets.

## 4. Conclusions

The aim of this paper was to determine a suitable adhesive for installation of a novel sensing sheet onto steel structural members subjected to cracking. Previous research has shown that for sensing sheets installed using a stiff adhesive (e.g., Araldite), the cracking in steel would induce immediate failure of individual sensors of the sheet. To address this challenge, three soft 3M glues were considered: DP100, DP105, and DP125.

The 3M DP125 adhesive was eliminated because the set time was too long to be useful in practical applications. Strain transfer tests were performed using strain gages to assess the quality of strain transfer of the two other glues. It was shown that while the Araldite adhesive transfers a large percentage of strain to the sensors, bonding with both the 3M DP100 and DP105 provides sufficient sensitivity to detect small strain changes, even in the non-damage range. No conclusive results were found to justify the preference between the DP100 or DP105 from the non-damage study, so crack opening damage tests were performed. 48-hour and 72-hour cure tests showed that the DP105 adhesive was able to detect strain changes for larger crack opening sizes compared to the DP100, so it was deemed more suitable for damage detection (cracking) in mechanically non-degrading materials (e.g., metals).

A sensing sheet prototype manufactured at Princeton University was then tested using the 3M DP105 adhesive. For each of the working individual sensors, apparent gage factors were determined by loading and unloading a thin aluminum beam with sand bottles, using theoretical strain values under Euler-Bernoulli beam theory. Uncertainties in the apparent gage factor values were found for both Araldite and 3M DP105-bonded sensor sheets. As expected, the apparent gage factors for the Araldite adhesive were higher than for the 3M DP105 adhesive. We know that an approximate gage factor for bonding a sensor sheet with 3M DP105 adhesive would allow one to use the sensor sheet to identify real strain values on structures.

The consistency between the strain transfer tests performed on regular strain gages and the tests performed on sensing sheets confirm that DP105 can be used for the installation of sensing sheets on steel structural members for crack detection and characterization purposes.

## Figures and Tables

**Table 1:** Apparent gage factor summary for Araldite and 3M DP105 adhesives ($\mu^{*}=$ mean, $\sigma^{*}=$ standard deviation).

	Araldite GF	3M DP105 GF
$\mu^{*}$	1.35	0.54
$\sigma^{*}$	0.240	0.128
